# Chloral Hydrate Enemata Preparatory to Ovariotomy

**Published:** 1879-10

**Authors:** C. H. Hughes

**Affiliations:** 1313 Chouteau Ave.; St. Louis


### ARTICLE VII.

### *Chloral Hydrate Enemata Preparatory to Ovariotomy.*

My opportunities having been large for the use of chloral, and having employed it for various purposes by enema, especially in delirium tremens and epilepsy (to assist the paroxysms of the latter), I offer the suggestion of chloral hydrate enemata as an immediate preliminary to the operation of ovariotomy.

Eighty grains dissolved in beef-tea, with a few drops of creosote added, may be safely and successfully used in epilepsia, puerperal eclampsia, and the persistent insomnia of obstinate delirium tremens, and I see no reason why, after the thorough clearing of the *primae viae* before proceeding to remove an ovarian tumor, it might not be the very best procedure to then administer a chloral enema, to put as effectual a quietus as possible on the bowels, and prevent the possibility of the annoying vomiting which so often follows the operation, and jeopardizes its success.

I think it quite possible for the operation to be safely begun on the patient while under the full influence of an eighty or hundred grain injection of chloral, and that the operation might proceed even to a completion of the operation in some instances, without having to resort largely to chloroform.

Vomiting during or immediately following ovariotomy, is a grave accident, and anything that promises to arrest it, ought to be tried, and in making trial of such remedies, if an agent be found that will put a quietus on puerperal and epileptic convulsions, and overcome the violent excitement and obstinate sleeplessness of acute insanity and *mania a potu*, such an agent should be tried. That remedy is hydrate of chloral.

In the use of chloral enemata, a single large dose should be given, and an interval of eight to twelve hours should be allowed to elapse before its repetition. This is the safe plan also when administering this valuable and potent agent *per orem*.

My experience embraces the administration of several hundred pounds of this drug in the last ten years without an accident traceable to its use. I have often given one drachm by the mouth, and not unfrequently one hundred grains *per rectum*, but have rarely ever repeated the former at less than eight hour intervals, or the latter at less than twelve.

C. H. Hughes, M.D.

St. Louis, Sept. 2, 1879, 1313 Chouteau Ave.

